# Decline of Persistent Jaundice in a Patient With Autoimmune Hepatitis and Vanishing Bile Duct Syndrome Treated With Elobixibat for Constipation

**DOI:** 10.7759/cureus.80021

**Published:** 2025-03-04

**Authors:** Tân Trần Thị, Norihiro Imai, Yosuke Inukai, Takashi Honda, Hiroki Kawashima

**Affiliations:** 1 Gastroenterology and Hepatobiliary Center, Bach Mai Hospital, Hanoi, VNM; 2 Department of Gastroenterology and Hepatology, Nagoya University Graduate School of Medicine, Nagoya, JPN; 3 Department of Gastroenterology, Tosei General Hospital, Seto, JPN

**Keywords:** autoimmune hepatitis, cholestatic jaundice, constipation, elobixibat, pbc-aih overlap syndrome, vanishing bile duct syndrome

## Abstract

We present the case of a 49-year-old woman with autoimmune hepatitis and persistent jaundice. On admission, pathology and laboratory results supported the diagnosis of autoimmune hepatitis-primary biliary cholangitis (AIH-PBC) overlap syndrome with vanishing bile duct syndrome (VBDS). Standard treatment, including methylprednisolone pulse therapy, prednisolone, azathioprine, bezafibrate, and ursodeoxycholic acid, failed to resolve jaundice. In addition to jaundice, the patient also had constipation and regularly used magnesium oxide and sennoside. Notably, the addition of elobixibat, initially prescribed for constipation, resulted in a marked improvement in jaundice. This case highlights the diagnostic and therapeutic challenges of AIH-PBC overlap syndrome with VBDS, particularly in cases of refractory jaundice. The observed efficacy of elobixibat suggests that it may be a valuable adjunctive therapy for severe cholestasis. Further research is warranted to clarify its therapeutic potential and underlying mechanisms in similar cases.

## Introduction

Autoimmune hepatitis (AIH) presents with a variety of clinical phenotypes, in which the majority of patients respond to standard treatment with steroids and immunosuppressants [1]. Autoimmune hepatitis-primary biliary cholangitis (AIH-PBC) overlap syndrome is a rare condition that is commonly diagnosed by Paris criteria [2]. Recently, the Intractable Hepato-Biliary Disease Study Group consensus in Japan has issued guidelines for diagnosing this syndrome [3]. It is associated with a poorer treatment response and prognosis compared to the individual diseases [4]. Vanishing bile duct syndrome (VBDS) is an acquired but potentially serious form of chronic cholestatic liver disease, characterized histologically by intrahepatic bile duct reduction due to immunological injury [5]. Despite advancements in treatment, managing refractory jaundice remains challenging. Elobixibat, a first-in-class selective ileal bile acid transporter (IBAT) inhibitor, was introduced to treat chronic idiopathic constipation in Japan in 2018 [6]. It functions by reducing the active ileal reabsorption of bile acids, which increases the concentration of bile acids entering the colon and enhances colonic secretion and motility [7]. Although its mechanism of action implies a potential effect on the systemic bile acid pool, no reports have described improvement in jaundice with elobixibat administration. We report a case of AIH-PBC and VBDS with significant jaundice improvement while treating constipation with elobixibat.

## Case presentation

A 49-year-old woman was referred to our hospital with persistent jaundice. She had a history of liver dysfunction in annual health check examinations, but she did not seek further evaluation due to the absence of symptoms. Approximately nine months before her admission to our hospital, she consulted her local physician because of jaundice. Blood tests and liver biopsy were performed, and the results indicated a possible diagnosis of AIH. She was treated with prednisone (PSL) at a dosage ranging from 10 mg/day to 20 mg/day for approximately six weeks. Medications used with PSL during this time are lansoprazole tablet 15 mg and eldecalcitol capsule 0.75 μg.

However, no clinical improvement was observed during this period. Over six months, she discontinued PSL while maintaining ursodeoxycholic acid (UDCA). UDCA was initiated in the second month after local hospitalization with a dose of 600 mg, which was increased to 900 mg after two weeks. The blood test results obtained at a local hospital are presented in Table 1.

**Table 1:** Laboratory results at a local hospital

Serum Biochemistry	Value	Ref. Range	Serum Biochemistry	Value	Ref. Range
Total bilirubin (mg/dL)	4.9	0.2-1.2	Immunoglobulin G mg/mL)	1421	700-1600
Aspartate aminotransferase (U/L)	135	13-33	Immunoglobulin M (mg/mL)	194	50-269
Alanine aminotransferase (U/L)	122	6-27	Immunoglobulin A (mg/mL)	366	93-393
Alkaline phosphatase (ALP) (U/L) (IFCC)	647	38-133	Protein (g/dL)	7.8	6.7-8.3
Total cholesterol (mg/dL)	354	120-220	Albumin (g/dL)	2.8	4.1-5.1
Triglyceride (mg/dL)	88	30-130	LDL-Cholesterol (mg/dL)	141	0-140
HDL-Cholesterol (mg/dL)	135	≥40			

Physical examination revealed yellowing of the ocular conjunctiva and marked yellowing of the skin, with a height of 149 cm, weight of 46 kg, body mass index of 22.1, and normal vital signs. Additionally, she reported chronic constipation despite long-term treatment with magnesium oxide and sennoside. There was no medical or family history and no history of drug and alcohol abuse.

The blood test results obtained on admission are presented in Table 2. Viral serology was negative for hepatitis A, B, and C, Cytomegalovirus, and Epstein-Barr virus. Antibodies related to AIH were positive for an antinuclear antibody (ANA) speckled pattern and anti-smooth muscle antibodies at a titer of 1:320 and 1:20, respectively. Additionally, anti-mitochondrial antibodies (AMAs) were positive at titers of 1:20 while the anti-mitochondrial AMA-M2 antibody (AMA-M2) was positive. Along with elevated liver and biliary enzyme levels, the patient also had a significantly increased total bilirubin level. The platelet count was normal, and the Child-Pugh score was A (5), indicating no evidence of significant hepatic dysfunction or liver fibrosis.

**Table 2:** Laboratory results on the day of admission

Serum Biochemistry	Value	Ref. Range	Antibody Serology Tests	Value	Ref. Range
Total bilirubin (mg/dL)	9	0.4-1.5	Anti-U1 ribonucleoprotein antibodies	Negative	Negative
Direct bilirubin (mg/dL)	6.1	0-0.2	Anti–Sjögren's syndrome-related antigen A antibodies	Negative	Negative
Aspartate aminotransferase (U/L)	101	13-30	Anti–Sjögren's syndrome-related antigen B antibodies	Negative	Negative
Alanine aminotransferase (U/L)	75	7-23	Anti-topoisomerase I (anti-Scl-70) antibody	Negative	Negative
Alkaline phosphatase (ALP) (U/L)	1031	106-322	Speckled pattern of antinuclear antibodies	Positive (1:320)	Negative
ALP 1 (%)	17.4	0-5.3	Anti-mitochondrial M2 antibody	Positive	Negative
ALP 2 (%)	62.1	36.6-69.2	Anti-smooth muscle antibodies	Positive (1:20)	Negative
ALP 3 (%)	18.3	25.2-54.2	Anti-liver-kidney microsome 1 antibody	Negative	Negative
ALP 5 (%)	2.2	0-18.1	Immunoglobulin M (mg/mL)	194	50-269
ɣ-Glutamyl transpeptidase (U/L)	563	9-23	Immunoglobulin A (mg/mL)	366	93-393
Protein (g/dL)	7	6.6-8.1	Viral Tests		
Albumin (g/dL)	2.8	4.1-5.1	Human Immuno-deficiency Virus antibody	Negative	Negative
Ammonia (NH3) (g/dL)	51	38-70	Hepatitis B surface antigen (IU/mL)	0.02	≤0.05
C-reactive protein (mg/dL)	0.71	<0.14	Hepatitis B core antigen (S/CO)	0.1	≤1
Creatinine (mg/dl)	0.42	0.46-0.79	Hepatitis C virus antibody (S/CO)	0.1	≤1
Cholesterol (mg/dL)	790	142-248	Epstein-Barr Virus IgM antibody	Negative	Negative
Triglyceride (mg/dL)	290	30-117	Cytomegalovirus IgM antibody	Negative	Negative
HDL-Cholesterol (mg/dL)	12	48-103	Cytomegalovirus IgG antibody	Positive	Negative
LDL-Cholesterol (mg/dL)	178	65-163	Treponema pallidum hemagglutination assay	Negative	Negative
Immunoglobulin G (mg/mL)	1509	861-1747	Hepatitis A antibodies IgM	Negative	Negative
Endocrine tests			Hematology		
Thyroid-stimulating hormone (TSH) (μIU/mL)	0.35	0.35-4.94	Total leukocyte count (/µL)	6.5	3.3-8.6
Free triiodothyronine (FT3) (pg/mL)	2.69	1.68-3.67	Lymphocytes (%)	27.1	14-55
Free thyroxine (FT4) (ng/dL)	1.01	0.7-1.48	Hemoglobin (g/l)	10	11.6-14.8
Hematology			Red blood cell count (x10^4^/µL)	3.21	3.86-4.92
Prothrombin time-international normalized ratio	1.09		Platelet count (x 10^3 ^ /µL)	406	158-348
Activated partial thromboplastin time (%)	61.5	80-120	Prothrombin time (%)	82.6	80-120
Day	Immunoglobulin G (mg/mL) (861-1747)	ALP (U/L)	Total cholesterol (mg/dL)		
Day 0	1509	1031	790		
Day 25	784	991	-		
Day 32	764	994	-		
Day 39	766	1048	-		
Day 59	726	1406	-		
Day 79	731	1784	-		
Day 107	712	1471	810		
Day 155	752	1244	506		

**Figure 1:**
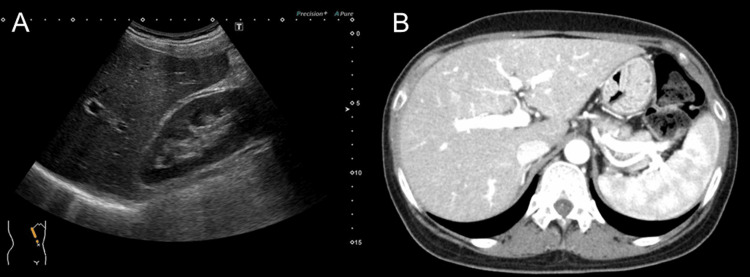
Findings from imaging studies (A) Ultrasonography. (B) Contrast-enhanced computed tomography. They showed no abnormalities in the liver morphology or hepatorenal echo contrast.

**Figure 2:**
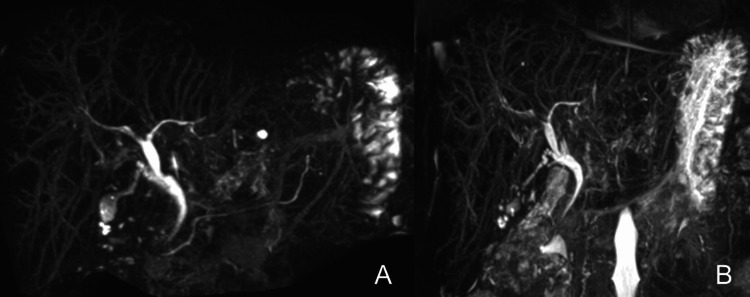
Comparison of magnetic resonance cholangiopancreatography (A) Magnetic resonance cholangiopancreatography (MRCP) performed five months prior to admission and (B) MRCP obtained upon admission revealed no abnormalities in bile duct structure and no significant changes over the five-month interval.

The imaging findings are shown in Figures 1, 2. 

Neither contrast-enhanced computed tomography nor magnetic resonance cholangiopancreatography revealed any defects, obstructions, or dilatations of the intrahepatic or extrahepatic bile ducts. Ultrasound images showed hepatomegaly, splenomegaly, and a smooth liver surface, with only a slight increase in liver parenchymal echogenicity.

**Figure 3:**
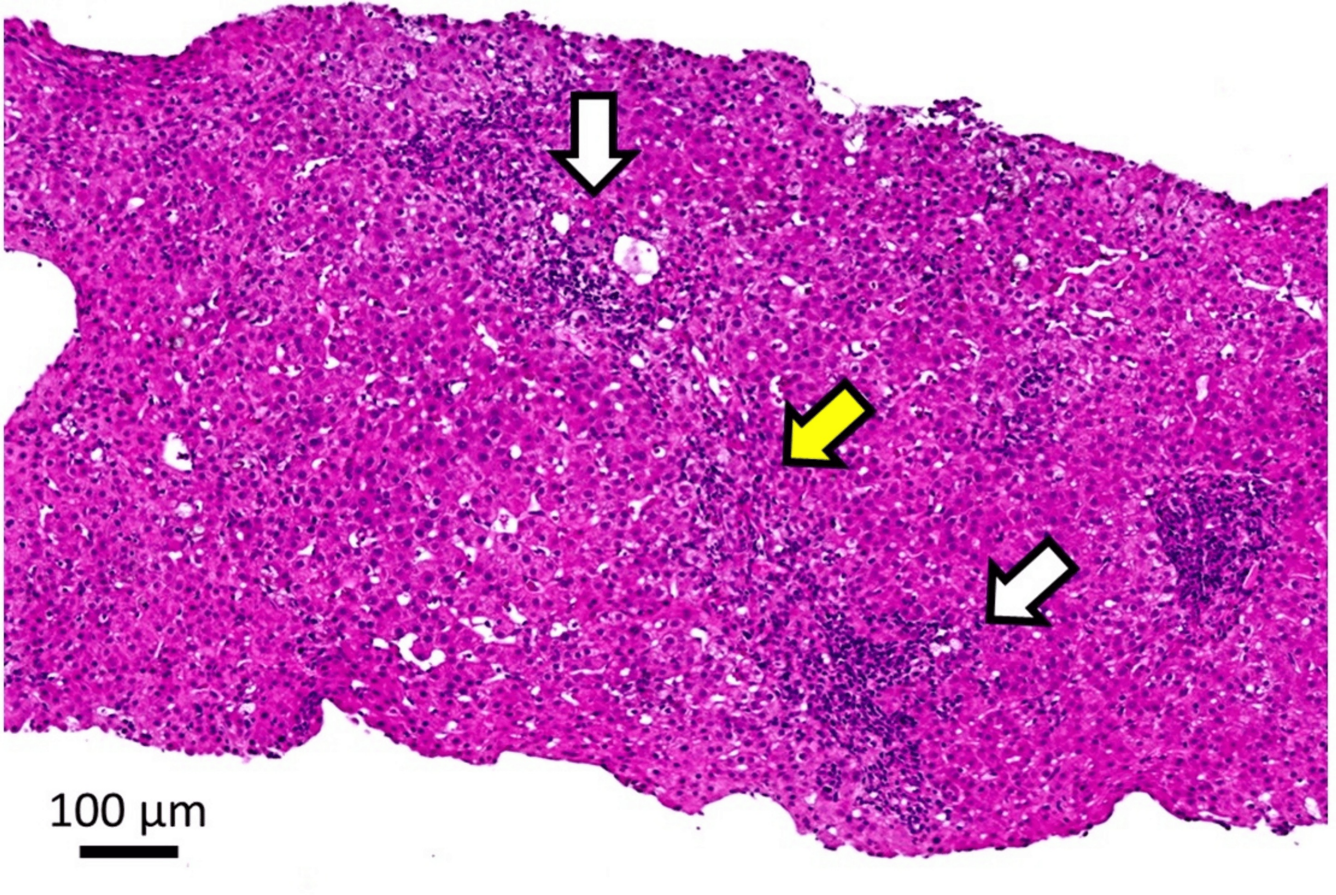
Histological evaluation of the first liver biopsy Hematoxylin and eosin (H&E) staining, original magnification X 200. Loss of interlobular bile ducts (white arrows) and interface hepatitis (yellow arrow).

A retrospective evaluation of the initial liver biopsy, conducted seven months before admission, revealed minimal fatty deposits, bile plugs in the bile ducts, lymphocytic portal inflammation, and mild fibrous enlargement. These findings were classified as grades A1 and F1. Although the patient tested positive for AMA-M2, the initial liver biopsy showed no findings specific to primary biliary cholangitis (PBC) but did reveal bile duct loss. The findings from the first liver biopsy are shown in Figure 3.

**Figure 4:**
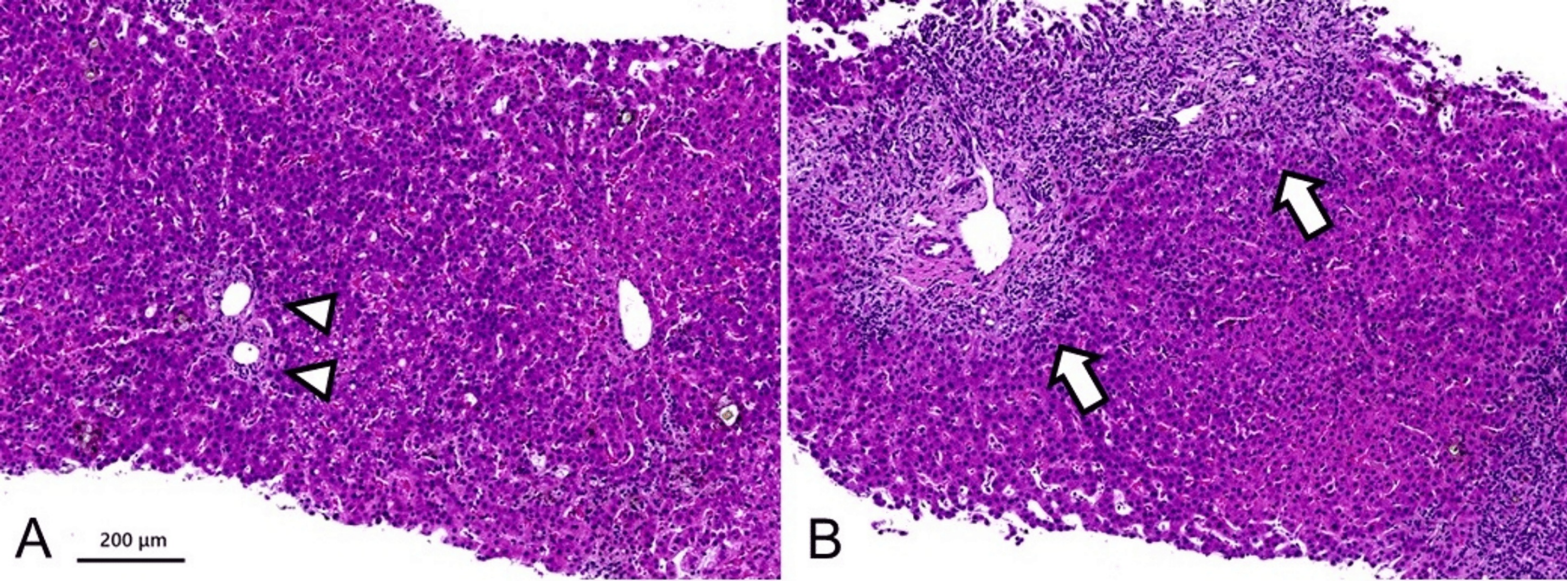
Histological evaluation of the second liver biopsy Hematoxylin and eosin (H&E) staining, original magnification X 100. (A) Loss of interlobular bile ducts (arrow heads) and cholestatic hepatocytes. (B) Inflammation in the form of lymphocytes, interface hepatitis (arrows), hepatocellular damage, spotty necrosis, and rosette formation.

A second liver biopsy was performed on the second day of admission. The findings from this liver biopsy are shown in Figure 4, highlighting the key pathological changes observed in liver tissue.

**Figure 5:**
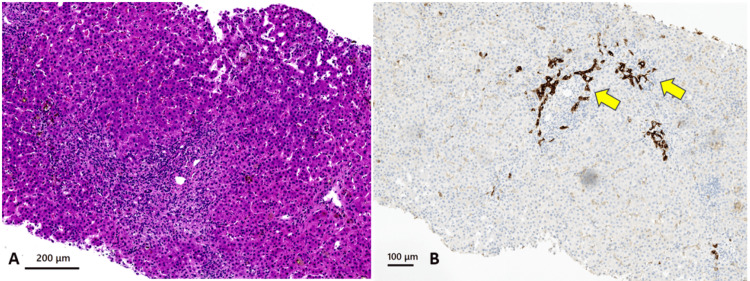
Histological evaluation of the second liver biopsy with CK19 immunostaining (A) Hematoxylin and eosin (H&E) staining, original magnification X 100. (B) CK 19 staining, original magnification X 200. A widened portal tract shows chronic inflammation and a ductular reaction (CK19 positive) but no native bile duct (yellow arrows). Bile plugs were observed around the portal tract.

The liver parenchyma exhibited a pattern of chronic inflammation with interface hepatitis, accompanied by scattered spotty necrosis, hepatocyte rosettes, and ductopenia in the portal areas. Cytokeratin 19 staining demonstrated pseudo biliary duct proliferation and unclear bile duct presence, suggesting bile duct loss (Figure 5).

Additionally, scattered areas of lymphocyte infiltration were observed, sometimes accompanied by neutrophils and plasma cells. No significant fibrosis was observed. These findings were classified as grades A1-2 and F1.

Based on these findings, the patient was diagnosed with overlap syndrome of AIH and PBC, accompanied by VBDS. She received intravenous methylprednisolone (mPSL) pulse therapy (1,000 mg/day) for three days, followed by oral PSL starting at 40 mg with gradual tapering and dose adjustments in the following two weeks. Subsequently, bezafibrate 400 mg/day was added after one week, and azathioprine (AZA) was introduced two weeks after PSL at an initial dose of 25 mg for one week before increasing to 50 mg. UDCA was administered at a dose of 900 mg/day from the start of treatment.

**Figure 6:**
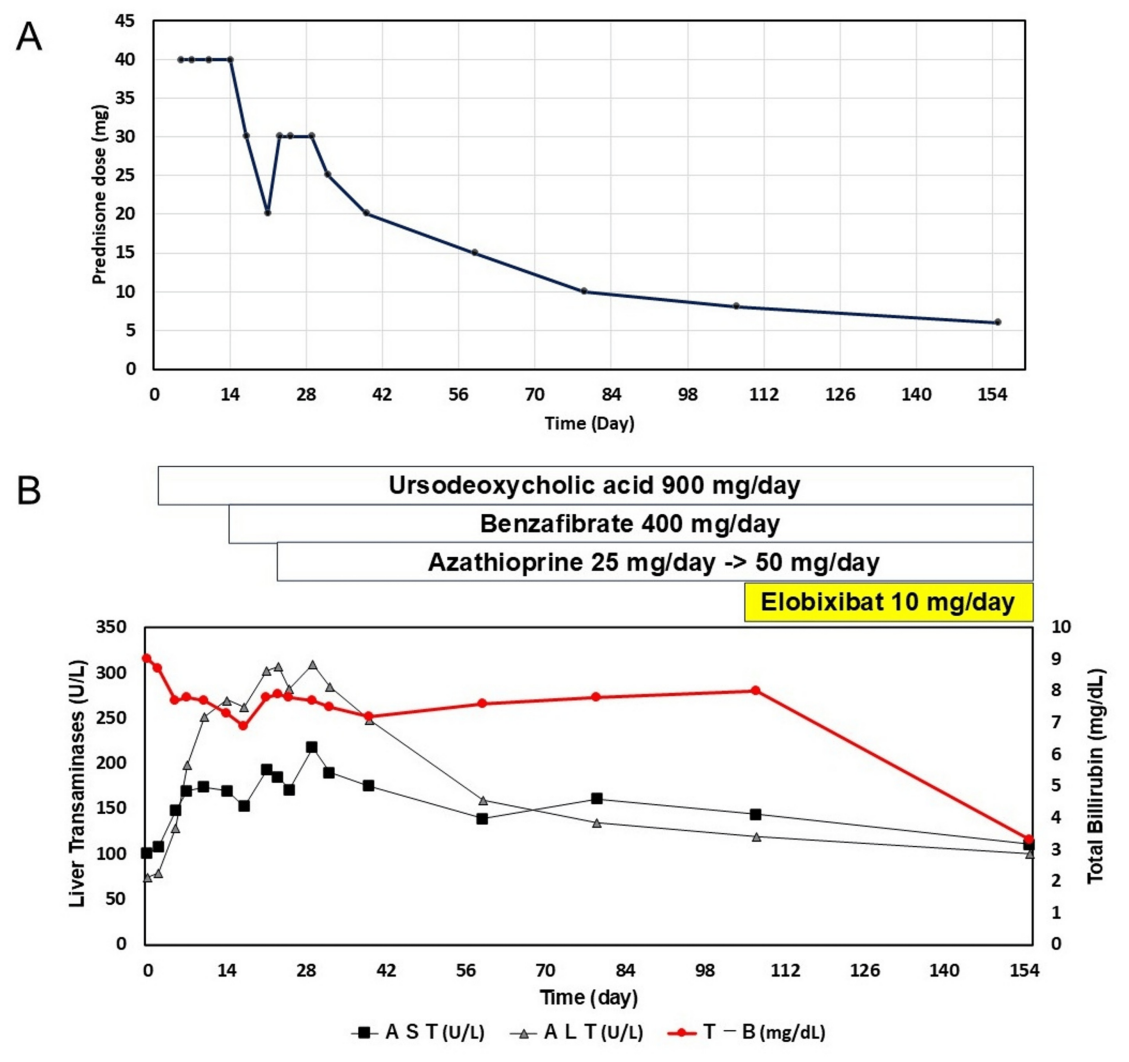
Changes in total bilirubin and liver transaminase levels (A) Corticosteroid therapy and tapering dose. (B) Bilirubin levels improved marginally with combination therapy, with significant improvement observed seven weeks after the addition of elobixibat. Liver enzyme concentrations tend to increase in the first four weeks and then decrease gradually. AST: aspartate aminotransferase, ALT: alanine aminotransferase, T-B: total bilirubin.

After AZA was added, liver enzyme levels began to decline, but severe jaundice persisted and exhibited a gradual worsening trend. Because her chronic constipation responded poorly to magnesium oxide and sennoside, she was prescribed elobixibat. Approximately 15 weeks after the mPSL pulse, during treatment with 8 mg PSL + 50 mg AZA + 400 mg bezafibrate + 900 mg UDCA, 10 mg elobixibat hydrate before breakfast was started. Surprisingly, a significant improvement in jaundice was observed seven weeks after adding elobixibat hydrate. The changes in total bilirubin, liver transaminase levels, and PSL dose are shown in Figure 6.

## Discussion

Here, we discuss a patient diagnosed with overlap syndrome of AIH and PBC accompanied by VBDS. Biliary obstruction was excluded through imaging studies, and based on blood test results, elevated alkaline phosphatase (ALP) levels, and positive AMA-M2, the diagnostic criteria for PBC were met [8,9]. This suggests a discrepancy between the initial liver biopsy histopathological features and the diagnosis. Additionally, there was a lack of response to prolonged UDCA therapy. We cannot rule out the possibility that minimal biliary changes in PBC were not observed in the initial liver biopsy specimen [10-12]. Therefore, a second liver biopsy was proposed with the expectation of identifying features that would support a definitive diagnosis.

After the second liver biopsy, the histopathological features along with high-titer positive ANA are consistent with the diagnosis of AIH. Ductopenia and reactive bile duct proliferation were noted in the cholestatic liver tissue, leading to a diagnosis of VBDS. While no specific features of PBC were found, an overlap syndrome diagnosis was established based on the Paris criteria and the Intractable Hepato-Biliary Disease Study Group consensus in Japan [2,3].

VBDS is an acquired disorder that often occurs secondary to many diseases [13]. Although its pathogenesis is unclear, bile duct destruction is attributed to an immune response predominantly mediated by T lymphocytes against the biliary epithelium [14]. Thus, case reports to date have frequently indicated that VBDS manifests in the context of PBC, drug-induced liver injury, Hodgkin's lymphoma, and hypersensitivity syndrome [13,14]. Both AIH and AIH-PBC overlap syndrome with VBDS are rare combinations, and the underlying mechanisms behind them are poorly understood. Although PBC is one of the most common causes of VBDS [15], the absence of other biliary injuries complicates the determination of the underlying cause of VBDS in this patient.

AIH-PBC overlap is treated with UDCA in addition to prednisone monotherapy or prednisone and azathioprine therapy [2]. Combination therapy has been shown to improve biochemical tests, stabilize fibrosis, and preserve five-year transplant-free survival [16]. Additionally, combination therapy is superior to prednisone alone and UDCA alone, as determined by the meta-analysis [4]. Returning to the case, the patient did not respond to prior corticosteroid and UDCA therapy. Consequently, a combination therapy including corticosteroids, UDCA, and bezafibrate was employed, and AZA was also administered during corticosteroid tapering. UDCA is widely used in the treatment of VBDS in drug-induced liver injury and shows benefits in improving liver damage. The recovery times for alkaline phosphatase and total bilirubin levels vary from several months to several years, and most of these conditions progress slowly and lead to cirrhosis, ultimately requiring liver transplantation [15,16].

The patient exhibited a gradual improvement in liver transaminases and immunoglobulin G levels over four weeks, which continued thereafter. However, total bilirubin levels remained persistently elevated. The indicators necessitating prolonged treatment and close monitoring include total bilirubin levels exceeding 2 mg/dL after three months of treatment, as well as the presence of ANA and AMA positivity [17]. Nearly unchanged total bilirubin levels suggest a potentially prolonged disease course [17]. Graf et al. noted that even with biochemical response, disease progression was also observed in patients treated with combination therapy at the end of follow-up compared to baseline [18]. The total number of patients with fibrosis and cirrhosis increased at the end of treatment, suggesting that despite normalization of liver enzymes, complete remission of histological inflammatory activity cannot be assumed in all patients [18]. Likewise, Park et al. showed that the overlap syndrome patients showed a lower rate of remission to UDCA and steroid combination therapy and significantly shorter time-to-progression of liver disease than that of the AIH patients (one-year and five-year progression rate, 0.0% and 34.4%, respectively) than that of the patients with AIH (one-year and five-year progression rate, 4.7% and 9.8%, respectively, p=0.013) [19].

Cholestasis can alter the concentration of bile acids in the intestines and circulation. Elobixibat reduces bile acid reabsorption in the ileum, increasing the amount of bile acids reaching the colon [20]. Incidentally, alongside the improvement in constipation after seven weeks of elobixibat administration, a significant reduction in jaundice was witnessed in the patient. Although its mechanism of action suggests that it may affect the systemic bile acid pool, no reports have described an improvement in jaundice with elobixibat administration. Due to its mechanism of action, elobixibat may have limited efficacy in patients with biliary obstruction or decreased bile acid secretion. Additionally, its IBAT inhibitory effect is believed to inhibit the reabsorption of bile acid preparations, such as UDCA. However, the results of this case suggest that administering elobixibat may be beneficial for patients with prolonged jaundice accompanied by constipation. In addition, hyperlipidemia, primarily characterized by elevated cholesterol levels, was observed in our patient. The mechanism of hyperlipidemia in VBDS has not been fully understood. It has been suggested that cholestasis might affect cholesterol metabolism. Despite treatment with bezafibrate, blood cholesterol levels did not improve for 10 weeks. A significant reduction in cholesterol levels was also observed following a substantial decrease in total bilirubin levels, further supporting the association between cholestasis and hypercholesterolemia. 

## Conclusions

In summary, the patient presented with seropositive AIH-PBC overlap syndrome combined with VBDS, showing inadequate response to steroids, AZA, UDCA, and bezafibrate. A significant improvement in total bilirubin levels was observed with the use of elobixibat hydrate for the treatment of constipation. The actual efficacy of elobixibat hydrate in AIH-PBC overlap syndrome and VBDS remains unknown, necessitating further observation of clinical progression and additional studies.
